# The changes, formation and public policy measures of mental health in Chinese youth

**DOI:** 10.3389/fpsyg.2025.1583594

**Published:** 2025-04-17

**Authors:** Yuanchao Wang, Guifeng Tan, Tao Zhu

**Affiliations:** ^1^School of Philosophy and Social Development, Shandong University, Jinan, China; ^2^National Academy of Chinese Modernization, Chinese Academy of Social Sciences, Beijing, China

**Keywords:** youth, mental health, confidence in development, change, formation mechanism

## Abstract

Confidence is an important indicator of individuals’ mental health. Taking confidence as an indicator, this paper uses CGSS2010-2021 data to analyze the latest situation, changing trend and formation mechanism of mental health in Chinese youth. The results show that firstly, Chinese young people have strong confidence in their future development in the new era, and their status expectations lean toward the upper and middle classes are dominant. Secondly, under the background of continuous economic growth and continuous improvement of objective status, youth confidence in development has not been significantly improved, but slightly decreased, resulting in a “paradox of confidence in development.” Thirdly, the composition effect (the changes in formation mechanism of status expectations) caused the above paradox. The positive influence of objective social status significantly reduced, and the positive influence of income gap disappears or turns negative. Youth confidence in development no longer depends on “who I am,” but more on the “macro environment.” Such changes offset the gains brought by economic growth and objective status improvement, leading to a fall rather than a rise in youth confidence in development.

## 1 Introduction

Mental health is the cornerstone of a happy life. As an important reflection of mental health, confidence always plays a key role in enhancing psychological resilience and decreasing mental health problems. In recent years, China has made remarkable progress in its economic and social development. Facing changes unseen in a century, China’s development is also facing severe challenges: Economic reform has entered a critical stage, with the income gap remaining high and the arguments in class solidification is rampant. In particular, the occurrence of international political conflicts and major public health events has sharply increased social risks and uncertainties. The weakening of expectations and the lack of confidence have not only become important issues that limit economic growth and social development, but also become obstacles of individual and social mental health development. Especially among the youth, the increase of social uncertainty is accompanied by the increasingly severe employment situation, which shakes youth confidence in the future development. Whether it is the bewildered sentiments such as “lying flat” and “Buddha-like,” or the phenomena of “involution” and “civil servants craze,” all are intuitive reflections of the issue.

In academic research, the aforementioned issue falls within the scope of studies on confidence in development. Confidence in development is a subjective reflection of individual mental state. It is an evaluation of self-development prospects for a period of time in the future based on real-life conditions (Cong, 2013). The level of people’s confidence in development can reflect the quality of public mental health from a long-term perspective, which has unique theoretical significance in sociological research. From the perspective of theoretical logic, the structural contradictions and tensions of society can be reflected not only by objective conditions such as social position, income and education level, but also by people’s subjective feelings or social attitudes: The former reflects the distribution issue under institutional design, the latter reflects the degree of public acceptance of institutional design or distribution structure (Li and Wei, 2013), which is the direct factor that triggers structural tensions and conflicts. Therefore, in the context of the rapid transformation of Chinese society, studies of social psychology/attitudes have attracted more and more attention (Li and Wang, 2020). Happiness, fairness, trust, including confidence in development, which are concerned in academic research, belong to the scope of social psychology/attitudes studies.

However, existing studies pay more attention on the overall social psychology/attitudes or specific issues. Research involving confidence in development is relatively scarce, and its unique academic value and application value have not been thoroughly elaborated. Firstly, confidence is an important indicator of social mentality, which reflects the overall mental health of society and serves as a “social barometer.” Different from studies focusing on people’s perception of the current situation, the research on confidence in development is oriented toward the future. It reflects whether the social environment is stable and orderly, and whether social development conforms to public opinion from another dimension of time. It is an indispensable part of the research on social psychology/attitudes. The lack of confidence means that people are dissatisfied with the distribution of benefits and channels of social mobility, which will not only lead to social anxiety but also lead to structural social resentment and a deep-seated social crisis (Lei, 2015). Secondly, confidence in development is a significant reflection of individual mental health, which can profoundly affect current behavior and psychology (Li and Liu, 2012), and then affect the overall economic and social development: Positive confidence in development helps to improve people’s mental health and cultivate positive social attitudes. On the contrary, negative confidence in development means that there is no hope, which will not only lead to negative emotions such as anxiety and depression (Zhu, 2013), but also lead to a decline in the willingness of investment, consumption and struggle, limiting economic growth and social development. So, whether for individuals or countries, the worst thing is not facing difficulties in their development, but that people lose confidence and hope in their future development (Li and Wei, 2013).

Young people are the future and the most active power in society. However, during the life course, young people are at the intersection of various life tasks such as employment, purchasing a house, marriage and childbearing. The impact of social problems such as slowing economic growth, rising living costs, and widening wealth gaps on their living conditions is particularly pronounced. On the contrary, the middle-aged and elderly people have entered a stable period in their careers, families and mental well-being, and are also a generation that has experienced a large-scale upward social mobility. The impact of current social problems on their confidence may be relatively slight. Thus, compared with the middle-aged and elderly people, young people are more likely to lose confidence in the context of stress and social anxiety, which in turn damages their mental health, and they are also the main power of future economic and social development. It is obvious that youth confidence in development is more sensitive in evaluating the social situation and social contradictions, and has a more direct impact on economic growth and social progress. Under the background of increasing social risks and uncertainties, it is important and urgent to monitor the mental health of young people and boost their confidence in development.

China, at the core of the East Asian Confucian cultural sphere, places great emphasis on education and career success both at the individual and family levels. The high population density and the limited availability of resources have further intensified the competitive pressures faced by young people, leading the widespread prevalence of mental health issues. Unlike South Korea and Japan, China has undergone dual social transformations of systemic transition and modernization within just four decades. The sustained economic growth has once brought about a strong sense of confidence in development. But in the past decade, with the slowdown of China’s economic growth, the social issues of urban–rural inequality and the widening income gap have gradually become more prominent. In the complex social context, the issue of mental health in Chinese youth is in urgent need of resolution, holds unique research value and gradually attracted attention. Also, young people’s expectations of social prospects and personal development have gradually been valued. A number of relevant empirical studies have emerged, providing detailed discussions on the basic situation, formation mechanisms, and subsequent impacts of youth confidence in development, offering valuable theoretical insights for this paper (Liu, 2016; Lei, 2015; Su, 2019; Zhao and Yuan, 2019). However, there are still two significant limitations in these studies: Firstly, in terms of research questions, existing studies are static and based on a single point in time, failing to capture the dynamic changes of youth confidence in development during the process of social transformation. Secondly, in terms of mechanism explanations, existing studies are limited to the micro perspective, without sufficient attention paid to the impact of macro-social-environmental factors.

In summary, youth confidence in development is an important issue reflecting the mental health of contemporary youth. It has important theoretical significance and practical value in contemporary China, characterized by both urgency and uniqueness. However, existing studies on this issue are neither comprehensive nor sufficiently profound. From 2010 to 2020, China’s GDP growth rate declined year by year, and the level of social mobility also began to decrease (Li et al., 2018). Statements such as “class solidification” and “lying flat” have become more common in public opinion. It is urgent to boost youth confidence in development and promote youth mental health. Based on this, we use the data from the Chinese General Social Survey to measure the mental health condition of young people through the indicator of youth confidence in development, and comprehensively analyze the changing trend and formation mechanism of Chinese youth mental health over the past decade, so as to add historical and macro perspectives to this issue.

The specific empirical questions are as follows: In the face of the rapid changes in the macro social environment, what is the latest situation of youth confidence in development? What changes have taken place in youth confidence in development compared with the past 10 years? At the micro and macro levels, which factors will affect youth confidence in development? Have these factors themselves and their impact on youth confidence in development changed? If so, which change determines the changing trend of youth confidence in development?

## 2 Literature review

### 2.1 Existing mechanism explanation: micro perspective

How is youth confidence in development formed? Theoretically speaking, people’s imagination of the “future” is based on the construction of the “present.” Confidence in the future development is also closely related to the experience of the present. It is an evaluation of future development prospects made by members of society based on their current real-life experiences (Cong, 2013). So, how does the present experience shape the confidence in development? In this regard, existing studies mainly explain from the micro perspective, and there are two theoretical perspectives: objective structure theory and subjective interaction theory.

#### 2.1.1 Objective structure theory

Objective structure theory, also named “structural determinism” and “social fact theory,” perceives that people’s confidence in development is determined by their objective socioeconomic status. The reason is that the acquisition of dominant social status is not overnight but a gradual and cumulative process. Entering top middle schools has a significant positive impact on entering top universities (Liu, 2006). People’s first jobs have a significant gain in their final occupational status. Similarly, the current objective socioeconomic status is an important starting point for future development. The higher the objective social status is, the higher the confidence in development will be (Liu, 2016). Interviews with Shanghai youth found that differences in objective status can lead to differences in young people’s visions of the future (Su, 2019). Young people at the bottom of the social ladder with low income and unstable employment tend to lose confidence in their future development, while young people from the middle-and upper-class tend to be more optimistic about their future career development.

#### 2.1.2 Subjective interaction theory

Structure theory and interaction theory are a pair of basic theoretical paradigms in sociological theory. Different from structure theory, which emphasizes the influence of absolute positions and physical environments, interaction theory emphasizes the dynamic processes of interaction among individuals and groups. In the studies of confidence in development, subjective interaction theory starts from the individual dimension, using comparisons with close others as a mechanism, and emphasizes the decisive role of subjective social comparison in shaping confidence in development. People’s visions of the future are formed by their daily life experience, and the ubiquitous social comparison is one of the basic links in constructing human life experience. In life, people would like to constantly compare with their past experience or reference groups around them, and when there is a strong sense of relative deprivation in social comparison, it is easy to lose confidence in future development.

The reason is that when individuals are in an advantageous position in social comparison, they tend to have a stronger sense of self-efficacy. The high evaluation of self-ability leads them to believe that “where there is a will, there is a way,” thus they have higher confidence in future development. Conversely, when they are in a relatively deprived position in social comparison, due to self-attribution bias, they are more likely to attribute the cause of failure to an adverse external circumstance (Alves and Rossi, 1978), so the experience of frustration can make them more pessimistic about the social environment, and lose confidence in the future development. In the empirical research, Kaichun Lei found that young people who believe that their life has improved significantly and have a higher sense of social fairness, will have greater confidence in their future class status (Lei, 2015).

### 2.2 Supplementing theoretical basis: issues of change and macro perspective

Under the guidance of objective structure theory and subjective interaction theory, existing studies have made a detailed discussion on the basic situation, formation mechanism and subsequent influence of Chinese youth confidence in development. Firstly, Chinese youth are generally optimistic about the future development. For instance, Kaichun Lei found there are more than 70% of young people are confident to join in the middle and upper classes (Lei, 2015). Secondly, objective social status and subjective social comparison will significantly affect youth confidence in development. Young people with higher levels of education, income and occupational status tend to be more confident in their future development, whereas those with low sense of social fairness and strong sense of relative deprivation are more likely to lose confidence in their future development (Liu, 2016; Su, 2019; Zhao and Yuan, 2019). Lastly, the lack of youth confidence in development can lead to social issues such as declining fertility intention and insufficient willingness to consume (Zhou and Yu, 2015; Chen and Li, 2021).

Nevertheless, there are still two essential growth points in theory in this scope: issues of change and the macro perspective. First, in terms of research questions, existing studies are based on the static research of a single time point, which cannot reflect the changing process of youth confidence in development. Sociology came into being under the background of rapid social change in modern and contemporary times. It is one of the core theoretical propositions of sociology to understand the changing process of social structure, predict and change the direction of social change on this basis. But this profound social change is not only reflected in modes of production and political systems, but also in cultural concepts such as values and social attitudes. In the field of cultural concepts, confidence in development is a rare and future-oriented attitude indicator, which has unique academic value. At the same time, the dual social changes of modernization and market transformation are the most basic macro background for discussing issues in China (Cai et al., 2020). Therefore, the perspective of change is an important way to understand issues China and understand the direction of social development. In the context of rapid changes in the macro social environment, especially in the context of the slowdown in economic growth and the rise discussion of class solidification in the past decade, exploring the changes in youth confidence in development is of great significance for understanding the overall trend of social development and guiding the sound operation of the economy and society.

Second, in terms of mechanism explanations, whether it is objective structure theory or subjective interaction theory, it is a micro and individualized theoretical perspective, lacking theoretical concern about the macro social environment. The reason is that studies of social psychology/attitudes are closely related to psychology in terms of the theoretical origin, so there is a tendency of micro individualism to a certain extent, and the influence of macro social structure factors is neglected in theoretical explanations. In recent years, studies on social attitudes have gradually broken free from the limitations of the micro perspective, with increasing emphasis on macro-environmental theories. In fact, members of society do not live in a vacuum, but are deeply constrained by the macro-social structural factors in which they live, and the social environment will be differentiated due to time changes and space differences. Therefore, macro-environment theory is a theoretical perspective of change and regionalization. It perceives that macro-social structural factors have a significant impact on individuals’ subjective attitudes, which has been confirmed by numerous cutting-edge studies (Lindemann and Saar, 2014; Curtis, 2016; Chen and Fan, 2016; Li, 2021). It is a pity that these studies do not focus on the issue of youth confidence in development. Therefore, explaining the formation mechanism of youth confidence in development from the perspective of macro-structure theory is conducive to promoting the enrichment and development of relevant theories in this scope, so as to better understand, predict and boost youth confidence in development, and promote youth mental health and guide them toward positivity and goodness.

## 3 Hypotheses

### 3.1 The theoretical foundation of understanding youth confidence in development

Nowadays, the second decade of the 21st century has quietly passed, China’s social transformation and modernization process is still advancing rapidly, the macro social environment has undergone major changes, and socialism with Chinese characteristics has entered a new era. As a generation moving forward with the new era, what is the latest situation of youth confidence in development? How has it changed compared to 10 years ago? Based on existing studies, this paper proposes a more comprehensive research framework to answer this question.

The theoretical foundation for understanding youth confidence in development and its changes lies in taking into account both micro and macro perspectives, combining objective structure theory, subjective interaction theory as well as macro environment theory, and explaining confidence in future development through current life experience, that is, youth confidence in development is determined by objective socioeconomic status, subjective social comparison and macro social environment. In the macro environment theory, the economic development level, housing costs, income gaps and other factors are common social-structural factors that affect people’s subjective attitudes (Lindemann and Saar, 2014; Curtis, 2016; Chen and Fan, 2016; Li, 2021). These factors are also closely related to the current living conditions of young people, and have a potential impact on youth confidence in development from the theoretical logic.

Firstly, the economic development level may promote youth confidence in development, as economic development means a higher degree of modernization. The occupational structure based on the secondary and tertiary industries can provide more middle-class jobs, and the selection mechanism based on meritocracy is conducive to the career promotion of young people (Erikson and Goldthorpe, 1992). Therefore, when the economy develops and the industrial structure upgrades rapidly, young people will have more equitable upward mobility opportunities, resulting in stronger confidence in development. Therefore, we can put forward Hypothesis 1.1,

Hypothesis 1.1: The higher the economic development level, the higher youth confidence in development.

Secondly, housing costs may reduce youth confidence in development. Housing has many attributes such as residence, investment, wealth reproduction, and identity symbol. It is a necessary consumption in Chinese family life. If housing prices are too high in a society, it will bring great economic pressure to young people, crowding out their space for human capital investment and other identity consumption. As a result, young people lack the time to improve their self-ability and are excluded from the leisure activities associated with middle-class identity (Veblen, 2017). Ultimately, this leads to widespread social anxiety. Therefore, we can put forward Hypothesis 1.2,

Hypothesis 1.2: The higher the housing costs, the lower youth confidence in development.

Thirdly, income gaps may reduce youth confidence in development, because those who are in a dominant position in the social stratification tend to limit the out-group to high-quality resources and opportunities through social closure and social exclusion mechanisms (Parkin, 1974), so that the dominant social status can continue to be maintained and transmitted between generations, that is, the so-called phenomena like “class solidification” and “born with a silver boon.” In a society with a large income gap, young people are more likely to perceive barriers to upward mobility and experience intergenerational wealth transmission, which in turn leads to lower confidence in their future development. Based on this, we can put forward Hypothesis 1.3,

Hypothesis 1.3: The higher the income gap, the lower the confidence of youth in development.

It should be noted that income gaps may also increase youth confidence in development. Economist Hirschman found that in the early stage of rapid economic development, people tend to have a higher tolerance for income gaps, because people will have higher expectations of their future income due to the increase in others income (Hirschman and Rothschild, 1973). Hirschman vividly calls this the “tunnel effect”: Just like in a traffic-clogged tunnel, people will be happy because the next lane begins to move, because this means that the traffic jam in front is coming to an end.^1^ Similarly, during the period of rapid economic growth in China, some people who get rich first will make most people think that they can also benefit from future economic development, thus enhancing their confidence in development. Based on this, we can put forward Hypothesis 1.4,

Hypothesis 1.4: The higher the income gap, the higher youth confidence in development.

### 3.2 The changing direction of youth confidence in development

As mentioned above, youth confidence in development depends on their objective social status, subjective social comparison and macro social environment. Then, according to the changes in the above three factors in the past decade, the change direction of youth confidence in development can be predicted. Specifically, this paper proposes a pair of rival hypotheses.

#### 3.2.1 Positive hypothesis

As far as the macro environment is concerned, since 2010, China’s national economy has continued to grow steadily, and per capita GDP has maintained a medium-to-high rate of increase, and the economic development level in various regions has reached a new level. Even under the impact of the COVID-19 pandemic, China is still the only major economy that has achieved positive economic growth. By 2021, the gross domestic product of China exceeded the 11-billion-yuan mark, firmly remaining the world’s second largest economy.

With the increase of macroeconomic level, people’s objective socioeconomic status is also gradually improving. First, residents’ income has increased significantly, and their living standards have improved significantly. From 2010 to 2021,^2^ per capita disposable income increased from 12,520 yuan to 35,128 yuan, and the Engel’s coefficient also decreased from 33.4 to 29.8%. Second, the education level has continued to increase. According to the data from the Seventh National Population Census of China, there were 15,467 people with junior college degrees or above per 100,000 people in 2020, which was approximately 1.73 times that in 2010. Third, the industrial structure has been continuously upgraded, and the average occupational status has risen. The proportion of the added value of the tertiary industry in GDP increased from 44.2% in 2010 to 54.5% in 2020, and the proportion of the employed population in the tertiary industry also increased from 34.6 to 47.7%, which promoted the proportion of the middle class in the occupational structure.

In addition, in terms of subjective social comparison, under the background of rapid modernization and market transformation in China, the continuous upgrading of the industrial structure and the increasing popularity of meritocracy have promoted the absolute social mobility rate to maintain a growth momentum for decades (Li and Zhu, 2015; Li et al., 2018), it can be inferred that people’s perception of upward mobility is also gradually improving in the new era. At the same time, with the reform and opening-up entering a new stage, the phenomena of “the inversion of the traditional income hierarchy between mental and physical labor” and corruption in the early stage of reform have been effectively curbed. Derived from the era of “egalitarianism,” the dissatisfaction and resentment that were intensified by the social reality in the early stage of reform and opening-up have declined, and the acceptance of income gaps is increasing (Xu et al., 2020). The overall sense of social fairness of the public has also shown an upward trend (Li, 2019).

In summary, from 2010 to 2021, the development level of China’s macroeconomy continuously improved, and people’s income, education level and occupational status also gradually improved. The sense of relative deprivation perceived by subjective social comparison was reduced, so youth confidence in future development would also increase.

Therefore, we can put forward Hypothesis 2.1,

Hypothesis 2.1: From 2010 to 2021, youth confidence in development was increasing.

#### 3.2.2 Negative hypothesis

Since 2010, while China has made considerable progress in economic development, issues like the rising housing prices and the increasing pressure from living costs have remained persistent. Moreover, the problem of unequal wealth distribution has been worsening. These have caused widespread anxiety in society, which may inhibit youth expectations of their future status and offset the positive effects of other factors.

Since the State Council of the People’s Republic of China fully implemented the housing monetization reform in 1998, the average selling price of commercial housing in China has soared from 1,948 yuan/m^2^ in 2000 to 4,725 yuan/m^2^ in 2010, and reached 9,980 yuan/m^2^ in 2020, which is over five times the price in 2000 and more than double the price in 2010, with an average annual increase of 20.6%. The rise of housing prices is particularly exaggerated in big cities, even higher than the growth rate of personal income: In 2020, the average selling price of residential commercial housing in Beijing was 42,684 yuan/m^2^, equivalent to 248.8% of that in 2010. In the same year, Beijing’s per capita disposable income was 69,434 yuan, equivalent to 237.5% of that in 2010. Based on this situation, the price of an 80-square-meter house in Beijing in 2020 is equivalent to 49 years of income for an ordinary Beijinger. Such rapid growth in housing prices will inevitably undermine the positive effects of economic development, social status, and subjective comparisons on personal confidence in their personal development, thereby impairing youth confidence in their future development.

Since the 1980s, there has been a general trend of the widening income gap and increasing concentration of wealth in developed countries in Europe and the United States (Piketty, 2014). China is no exception. The widening income gap has become one of the most basic social problems since the reform and opening-up. Since 2008, China’s Gini coefficient has begun to decline, but it has also remained between 0.46 and 0.47, always above the social risk threshold (0.4). In the early stage of economic growth, rapid economic development and an appropriate income gap may lead to optimistic estimates of future development. However, as China’s GDP growth rate gradually slows down and economic development shifts to the new normal, the long-term effect of the “tunnel effect” will become apparent, that is, the long-term large income gap may reduce people’s confidence in development and trigger their anxiety and unease. In particular, it should be noted that the vested interest groups gradually formed in the past 40 years of the reform and opening-up, as their children gradually enter the youth stage, they carry out social closure and social exclusion through reproduction and ruling mechanisms, so as to realize the intergenerational transmission of their advantageous social status, which makes the relative social mobility rate begin to decline (Li et al., 2018). The various voices in society regarding “class solidification” are the direct manifestations of this issue. They also undermine youth confidence in their future development.

Therefore, we can put forward Hypothesis 2.2,

Hypothesis 2.2: From 2010 to 2021, youth confidence in development was decreasing.

## 4 Methods

### 4.1 Data source

We use the data from the Chinese General Social Survey (CGSS) in 2010, 2012, 2013, 2015, 2017, 2018 and 2021, which is conducted by the National Survey Research Centre at Renmin University of China. It is one of the earliest national, comprehensive and continuous academic survey projects in China. Referring to the *Medium and Long-term Youth Development Plan (2016–2025)* issued by the Central Committee of the Communist Party of China and the State Council of the People’s Republic of China, as well as the relevant definitions from the National Bureau of Statistics, this paper defines youth as those aged 14–35 years. (Note: The minimum age of the actual CGSS sample is 17 years.) After deleting missing values and singular values, a total of 14,751 valid samples are kept.

### 4.2 Measurements

#### 4.2.1 Dependent variable

Confidence in development is an evaluation of self-development prospects for a period of time in the future based on real-life conditions (Cong, 2013). It serves as a future-oriented indicator of social attitudes. As a positive emotional expression, confidence reflects people’s optimistic attitudes toward future life and is an important reflection of public mental health. Lack of confidence means that people are generally pessimistic and anxious about the future, which is a serious public mental health problem. Boosting confidence helps enhance psychological resilience, reduce negative emotional experiences, and plays an active role in helping individuals cope with difficulties and challenges. In particular, China is in an important period of social transformation. The uncertainties and risks in society make the issue of confidence in development urgently need to be focused on and addressed. Overall, unlike most social attitude indicators, confidence in development is future-oriented and has a stronger representativeness of mental health. It reflects the overall mental health of society more clearly through people’s simple imagination of the future. Therefore, we take youth confidence in development as an indicator of youth mental health. In empirical studies, researchers all use “5–10 years later” to represent the future, but there are still some differences in measuring self-development: Some use general terms like “good” and “poor” (Zhao and Yuan, 2019), or use multiple composite indicators such as income, housing, family and social status (Zhang et al., 2015), and some scholars focus on people’s confidence in social class and career development (Lei, 2015; Su, 2019).

Although social class is a single indicator, it can comprehensively reflect people’s position in the social stratification and is also a classic indicator for measuring social position in social stratification research. Thus, this paper measures youth confidence in development with future status expectations, taken from the question: “In our society, from 10 to 1, where 10 represents the highest class and 1 represents the lowest. Where do you think you will be in 10 years?” We regard it as a continuous variable. The higher the value, the higher the youth expectation of status, and the stronger their confidence in future development.

#### 4.2.2 Independent variables

This paper focuses on the influence of objective social status, subjective social comparison and macro social environment on confidence in development. Objective social status includes three variables: education, income and occupation. These are, respectively, measured by years of education, the logarithm of personal annual income and occupational status (1 = farmer, 2 = worker, 3 = non-manual worker, 4 = professional and technical personnel, 5 = manager), and personal income is adjusted according to the consumer price index.

Subjective social comparison includes the sense of upward mobility and social fairness. The sense of upward mobility is measured as the gap between “one’s current social class” and “the social class of their family at age 14.” These two questions are both based on personal subjective identity. The sense of social fairness comes from the question: “In general, do you think today’s society is fair?” Both variables can be regarded as continuous variables. The higher the value, the stronger the sense of upward mobility and social fairness, and the weaker the sense of relative deprivation.

In terms of macro social environment, it includes three variables: the economic development level, the house-price-to-income ratio, and the income gap. All these are measured at the provincial level of the sample.^3^ The data is sourced from the National Bureau of Statistics.^4^ Among them, the economic development level is measured as the per capita GDP of the current year. The house-price-to-income ratio refers to the ratio of the average selling price of commercial housing in the current year to the disposable income of residents in the same year. The higher the value of this indicator, the greater the pressure from living costs mainly on housing. The income gap is measured by the urban–rural income ratio and the individual income gap as indicators. The higher the values of these indicators, the greater the income gap. The urban–rural income ratio refers to the ratio of the disposable income of urban residents to that of rural residents in the current year. The personal income gap is measured by the Theil index. This index is a commonly used indicator for measuring the income gap and is calculated based on the personal annual income of the sample. The formula is:


$$T=\frac{1}{N}\sum_{i}\frac{y_{i}}{\bar{y}}\log\left(\frac{y_{i}}{\bar{y}}\right)$$

#### 4.2.3 Control variables

In addition to the independent variables, this paper also controls for the sample’s gender (0 = female, 1 = male), age, household registration (0 = agricultural, 1 = non-agricultural), marriage (0 = unmarried, 1 = married), political background (0 = non-CCP member, 1 = CCP member, where “CCP” stands for Chinese Communist Party), business type (0 = non-government funding support, 1 = government funding support), and population migration (0 = local population, 1 = floating population).

### 4.3 Models

OLS models are used to test the changing trend and the factors of youth confidence in development, and Oaxaca-Blinder decomposition is used to analyze the formation of this change. Oaxaca-Blinder decomposition is a classic method to decompose the difference in average wages and was proposed almost simultaneously by Ronald Oaxaca and Alan S. Blinder (Oaxaca, 1973; Blinder, 1973). This method was initially used to analyze income inequality between genders and races. It can decompose the difference in average wages into structural differences and coefficient differences (e.g., differences between men and women in educational attainment and the return on education). Later, it was introduced into the study on the changes of social attitudes (Li and Wang, 2020). This paper uses the period as the grouping variable. The changes in youth confidence in development are decomposed into structural differences and coefficient differences of factors such as objective social status, subjective social comparison, and macro social environment, in order to explore the impacts of the structure effect and the composition effect on these changes, and further determine how the trend of changes in confidence of youth in development is formed.

## 5 Results

### 5.1 Current situation of youth confidence in development in the new era

**Figure 1:**
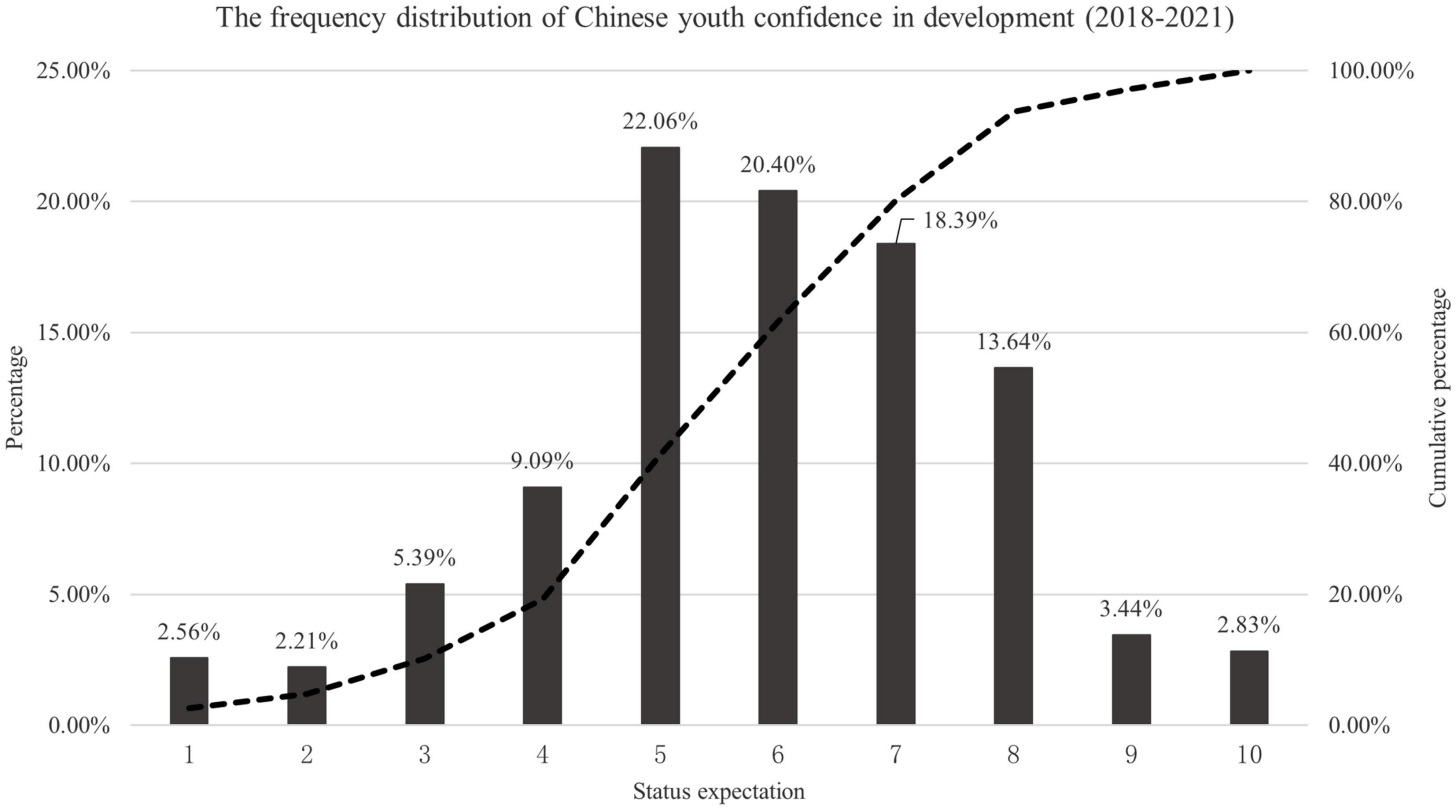
The frequency distribution of Chinese youth confidence in development (2018–2021).

Figure 1 is a frequency distribution graph of Chinese youth confidence in development from 2018 to 2021. The horizontal axis represents the expected social status after 10 years. The columns represent the percentage of expected status at different levels in the overall population (corresponding to vertical coordinates on the left). The dotted line represents the cumulative percentage of expected status at different levels (corresponding to vertical coordinates on the right). It can be seen that Chinese youth in the new era have strong confidence in future development and optimistic expectations for their personal status. Specifically, if the social class is divided into levels 1–10 from the lowest to the highest, with each score representing one class (the horizontal coordinates). Therefore, the average status expectation of Chinese youth from 2018 to 2021 was 5.892, and the overall distribution demonstrated a trend of peak shift. Over 80% of young people were confident that they would enter the middle and upper classes of society in 10 years. Nearly 60% of young people believed that they would be able to exceed the average level of social class (Level 5) in 10 years.

In terms of the structure, the above-average level of confidence in future development takes a dominant position. The proportion of those who believe they will be at levels 5–8 in 10 years was significantly higher than that of other youth. The respective proportions were 22.06, 20.40, 18.39 and 13.64%. And the total is 74.49%.

### 5.2 Changes in youth confidence in development

According to Table 1, since 2010, the level of China’s economic development has increased significantly, with the per capita GDP in each region rising significantly. Simultaneously, the objective social status of young people has also risen significantly. There have been significant increases in the average years of education, average personal annual income, and average occupational status. Additionally, from the perspective of subjective social comparison, although the sense of upward mobility has declined to some extent, the overall sense of social fairness has increased significantly.

**Table 1:** Descriptive analysis.

	2010–2013	2015–2017	2018–2021	Significance of difference	Direction
Confidence in development	**6.088**	**6.005**	**5.892**	0.000	−
Male	0.480	0.478	0.468	0.479	
Age	28.329	28.250	28.721	0.000	+
Non-agricultural	0.464	0.446	0.432	0.005	−
Married	0.692	0.625	0.597	0.000	−
CCP member	0.087	0.083	0.090	0.605	
Government funding support	0.202	0.186	0.193	0.117	
Floating population	0.217	0.267	0.269	0.000	+
Years of education	11.445	12.273	12.591	0.000	+
Occupational status	2.385	2.426	2.594	0.000	+
Log (personal annual income)	9.047	9.023	9.256	0.004	+
Sense of upward mobility	0.767	0.647	0.605	0.003	−
Sense of social fairness	2.870	3.120	3.246	0.000	+
Per capita GDP (10,000 yuan)	4.282	6.491	8.263	0.000	+
House-price-to-income ratio	0.359	0.325	0.323	0.000	−
Urban–rural income ratio	2.656	2.511	2.432	0.000	−
Personal income gap (Theil index)	0.604	1.018	1.053	0.000	+

The dependent variable is presented in bold to clearly highlight the changes in the confidence in development of Chinese youth, distinguishing it from the independent variables.

However, the average status expectation of Chinese youth was 6.088 in 2010–2013, but the average status expectations of Chinese youth were 6.006 in 2015–2017 and 5.892 in 2018–2021. In other words, Chinese youth confidence in future development has not increased, but has slightly declined. This means that the estimates of personal future development of young people in the new era may become relatively conservative, and this paper conducts more rigorous tests in subsequent models.

**Figure 2:**
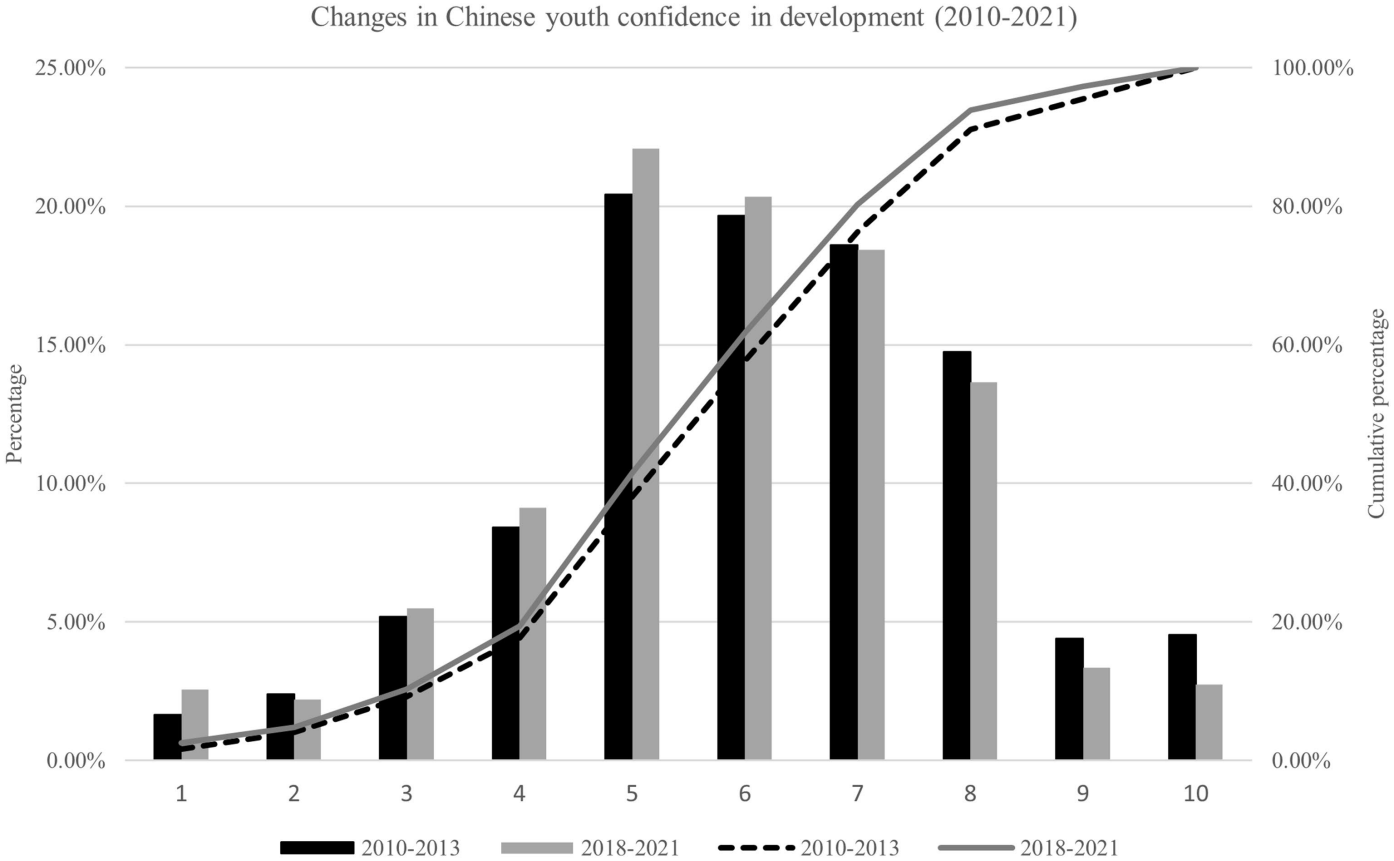
Changes in Chinese youth confidence in development (2010–2021).

Figure 2 is a comparative graph that compares the distribution of Chinese youth confidence in development in 2010–2013 with that in 2018–2021. The meanings of the horizontal axis, column, line, and ordinates on both sides are identical to those in Figure 1. The black column and black dotted line illustrate the situation in 2010–2013, and the gray column and gray solid line illustrate the situation in 2018–2021. According to Figure 2, compared with the situation in 2010, the dominant pattern of Chinese youth confidence in development has not changed. The above-average level of confidence in development has always remained dominant, and the overall distribution of youth confidence in development still leans toward the higher. The decline in the average confidence in development is mainly due to a slight decrease in the proportion of those who expect to enter the upper class of society.

### 5.3 Influence factors of youth confidence in development

In 1974, American economist Easterlin proposed a paradox regarding happiness, namely the “Easterlin Paradox”: An increase in personal income can significantly enhance happiness, but the growth of a country’s economy does not lead to an improvement in the happiness of its citizens (Easterlin et al., 2012). Not coincidentally, a similar “paradox of confidence in development” has emerged in the changes of Chinese youth confidence in development: While the national economy continues to grow at a medium-high speed, youth confidence in future development has not increased accordingly.

How can we explain this “paradox”? This paper argues that the changes in Chinese youth confidence in development are inevitably brought about by factors such as their objective social status, subjective social comparison, and macro social environment. However, in terms of the mechanism, these can be divided into two types of effects: Firstly, the structure effect, that is, the changes in the aforementioned factors themselves. Secondly, the composition effect, that is, the extent and even the direction of the impact of the aforementioned factors on the confidence in development.

To this end, this paper employs the annual linear regression model and the period-interaction model to test the changes in the formation mechanism of youth confidence in development across different periods. Table 2 shows the results. Model 1 is an OLS model based on the full samples. Model 2 uses a hierarchical linear model for robustness test. Models 3–5 reflect the situations of 2010–2013, 2015–2017, and 2018–2021, respectively. In the period-interaction model, the interaction terms consist of independent variables and year dummy variables. The significance of differences is determined by the significance level of these interaction terms.

**Table 2:** Regression model of youth confidence in development.

	Model 1	Model 2	Model 3	Model 4	Model 5	Significance of difference
	OLS	HLM	2010–2013	2013–2015	2018–2021	
Period						
2015–2017	−0.251^***^ (0.043)	−0.184^***^ (0.048)				
2018–2021	−0.445^***^ (0.052)	−0.369^***^ (0.066)				
Objective social status						
Years of education	0.060^***^ (0.006)	0.064^***^ (0.006)	0.072^***^ (0.009)	0.059^***^ (0.010)	0.051^***^ (0.011)	0.013
Log (personal annual income)	0.012^*^ (0.005)	0.011^*^ (0.005)	0.023^**^ (0.008)	0.008 (0.008)	−0.002 (0.009)	0.086
Occupational status	0.207^***^ (0.019)	0.201^***^ (0.019)	0.208^***^ (0.027)	0.218^***^ (0.036)	0.154^***^ (0.039)	0.464
Subjective social comparison						
Sense of upward mobility	0.098^***^ (0.006)	0.099^***^ (0.006)	0.231^***^ (0.013)	0.036^***^ (0.007)	0.229^***^ (0.019)	0.000
Sense of social fairness	0.035^***^ (0.008)	0.036^***^ (0.008)	0.183^***^ (0.021)	−0.003 (0.009)	0.165^***^ (0.033)	0.001
Macro social environment						
Economic development level	0.048^***^ (0.008)	0.042^**^ (0.013)	0.036^**^ (0.014)	0.038^*^ (0.015)	0.055^***^ (0.014)	0.306
House-price-to-income ratio	−1.615^***^ (0.256)	−0.737 (0.405)	−2.103^***^ (0.331)	−0.736 (0.549)	−2.147^***^ (0.627)	0.408
Urban–rural income ratio	0.364^***^ (0.050)	0.434^***^ (0.126)	0.467^***^ (0.068)	0.194^*^ (0.098)	0.089 (0.120)	0.003
Personal income gap	0.018 (0.029)	0.015 (0.031)	0.395^***^ (0.078)	0.013 (0.042)	−0.160^**^ (0.052)	0.000
Control variables	Included	Included	Included	Included	Included	Included
*N*	14,751	14,751	7,053	4,266	3,432	
adj. *R*^2^	0.076		0.122	0.063	0.088	

*t* statistics in parentheses ^*^ *p* < 0.05, ^**^ *p* < 0.01, ^***^ *p* < 0.001.

Controlling variables include gender, age, marriage, political background, business type, population migration, etc.

According to Model 1, in terms of the trend of changes, Chinese youth confidence in future development has significantly decreased year by year. Compared with the period of 2010–2013, the confidence of youth in 2015–2017 was 0.251 lower, and the confidence in 2018–2021 was 0.445 lower. The results of Model 2 are basically consistent with those of Model 1. It can be seen that over these 10 years, young people’s assessment of future development has become more conservative. Based on this, Hypothesis 2.1 can be rejected, and Hypothesis 2.2 can be verified.

In terms of the formation mechanism, or in other words, the influence factors, an individual’s objective social status, subjective social comparison, and macro social environment will all significantly affect their expectations of their future status. Firstly, having a high social status is conducive to enhancing youth confidence in development. After controlling for other variables, the longer the years of education and the higher the occupational status, the greater the confidence of youth in development. Secondly, subjective social comparison is beneficial for enhancing youth confidence in development. After controlling for other variables, the stronger the sense of upward mobility and social fairness, the greater the confidence in development of young people. Finally, macro social environment also has a significant impact on youth confidence in development. A developed regional economy as well as the income gap between urban and rural areas are conducive to enhancing youth confidence in development, while the high housing price will reduce it. Based on this, Hypotheses 1.1, 1.2, and 1.4 can be verified.

In terms of the formation mechanism, according to Model 3-Model 5, significant changes have occurred in the effect of each variable on youth confidence in development over the past decade. Firstly, the positive effect of objective social status on youth confidence in development has decreased significantly: The effect of education has declined by year. In 2010–2013, for every additional year of education, the average youth confidence in development increased by 0.072. But in 2018–2021, for every additional year of education, the average youth confidence in development only increased by 0.051. When the effect of income has disappeared, in 2010–2013, personal income had a significant positive effect on youth confidence in development. But in 2018–2021, the effect of personal income was not significant. According to the period-interaction model, the interaction term between years of education and years, as well as the interaction term between income and year are both significant, that is, the objective social status has a significant effect on the decline of youth confidence in development.

Secondly, the effect of subjective social comparison on youth confidence in development has also declined. Compared with 2010–2013, the positive effect of the sense of upward mobility and fairness had a significant decline in 2015–2017. Although the effect increased in 2018–2021, it did not reach the level in 2010–2013. According to the period-interaction model, the changes in the effect of the sense of upward mobility and social fairness are also significant.

Thirdly, the effect of macro social environment on youth confidence in development has changed significantly and is fairly complicated. The positive effect of the economic development level has increased. The effect coefficient of per capita GDP on youth confidence in development increased from 0.036 in 2010–2013 to 0.055 in 2018–2021. The negative effect of housing prices has undergone a “U-shaped” change. The effect had a significant decline in 2015–2017, and increased in 2018–2021, even higher than that in 2010–2013. But from the significance of the interaction terms, the changes of the above two items both failed to pass the significance test.

The effect of income gap on youth confidence in development has undergone a significant change. Compared with 2010–2013, the positive effect of urban–rural income ratio had a significant decline in 2015–2017, but it was not significant in 2018–2021. The impact direction of personal income gap has shifted from positive to negative. In 2010–2013, for every one-unit increase in personal income gap, youth confidence in development would increase by 0.395. But in 2018–2021, or every one-unit increase in personal income gap, youth confidence in development would decrease by 0.160 instead.

Standardized regression coefficients can reflect which factors have a more important impact on the confidence in development in different years. According to Table 3, since 2010–2013, the absolute values of standardized regression coefficients for objective social status and subjective social comparison have generally declined by year, which means that the importance of these two factors in determining youth confidence in development has diminished. Correspondingly, the importance of macro social environment in determining youth confidence in development has increased, and this is mainly reflected in the economic development level and housing prices. The standardized regression coefficients for these two factors increased from 0.041 and 0.081 in 2010–2013 to 0.114 and 0.102 in 2018–2021, respectively. They have thus become the two most important variables in shaping youth confidence in development, aside from the sense of upward mobility.

**Table 3:** Standardized coefficients of the regression model for youth confidence in development (absolute value).

	2010–2013	2013–2015	2018–2021
Objective social status			
Years of education	0.138	0.115	0.101
The logarithm of personal annual income	0.036	0.017	0.004
Occupational status	0.105	0.106	0.077
Subjective social comparison			
Sense of upward mobility	0.209	0.076	0.195
Sense of social fairness	0.099	−0.006	0.083
Macro social environment			
Economic development level	0.041	0.068	0.114
House-price-to-income ratio	0.081	0.034	0.102
Urban–rural income ratio	0.095	0.034	0.015
Personal income gap	0.057	0.005	0.057
Control variables	Included	Included	Included

Controlling variables include gender, age, marriage, political background, business type, population migration, etc.

To sum up, compare with the first decade in the 21^st^ century, the formation mechanism of youth confidence in development in the new era has undergone significant changes. The main manifestations are as follows: First, the effect of objective social status has been undermined. It is no longer the dominant factor of confidence in development. The gains of confidence in development derived from education and income has been significantly reduced. Second, the effect of macro social environment has intensified and has become one of the dominant factors in determining confidence in development. In particular, the gain in confidence derived from the economic development level has significantly increased. Third, the effect of income gap has shifted from positive to negative, and the long-term effect of the “tunnel effect” has begun to emerge.

The changes in the formation mechanism of youth confidence in development can help us explain the “paradox of confidence in development.” On the one hand, from 2010 to 2021, although the level of education and income of Chinese people improved continuously, an individual’s objective social status is no longer the main factor in determining confidence in development. Thus, the benefits it brings are limited. On the other hand, despite the increasing economic development level and its growing influence, the negative shift in the impact of income gap has significantly reduced youth confidence in development. The combined effect of these two factors has resulted in youth confidence in development in the new era not only failing to increase significantly, but also experiencing a slight decline.

### 5.4 Mechanism decomposition of changes in youth confidence in development in the new era

As mentioned before, Chinese youth confidence in development has significantly decreased in the past decade, and the confidence in development is determined by objective social status, subjective social comparison and macro social environment. Since 2010–2021, the objective social status of Chinese youth has improved significantly. Years of education, occupational status and personal income have increased significantly, the subjective social comparison of young people and macro social environment they are in have also undergone significant changes. These changes in the influence factors themselves can be named as “structural changes” or “distributional changes,” and the effect of these changes on the changing trend of youth confidence in development is the “structure effect.”

At the same time, according to Tables 2, 3, the formation mechanism of Chinese youth confidence in development has undergone a significant change in the past decade: The degree and direction of the impact as well as the relative importance of objective social status, subjective social comparison and the macro social environment all have altered. These changes can be named as the “coefficient changes,” and the effect of coefficient changes on the changing trend of youth confidence in development is the “composition effect.”

OLS model can help us analyze the changes in the formation mechanism of youth confidence in development since 2010, and it reflects the explanatory power of the composition effect on the changes in youth confidence in development in the new era. However, this model cannot distinguish structure effect and composition effect. Therefore, we use the method of Oaxaca-Blinder decomposition to analyze the formation mechanism of youth confidence in development in the new era specifically, and the results are shown in Table 4. Then, this paper is going to discuss how the objective social status, subjective social comparison and macro social environment influence the changes in youth confidence in development, respectively.

**Table 4:** The mechanism decomposition of the changes in youth confidence in development.

Total variation of youth confidence in development	Structure effect		Composition effect		Total effect
	Difference value	Contribution rate	Difference value	Contribution rate	Contribution rate
Control variables	Included	Included	Included	Included	Included
Objective social status					
Years of education	0.072^***^	36.67%	−0.261	−133.4%	−96.68%
Personal income	0.002	0.940%	−0.254^*^	−129.8%	−128.8%
Occupational status	0.041^***^	20.73%	−0.154	−78.68%	−57.95%
Subjective social comparison					
Sense of upward mobility	−0.037^***^	−18.70%	−0.003	−1.550%	−20.24%
Sense of social fairness	0.064^***^	32.80%	−0.063	−32.03%	0.770%
Macro social environment					
Economic development level	0.192 ^***^	98.21%	0.121	61.89%	160.1%
House-price-to-income ratio	0.071^***^	36.08%	−0.020	−10.29%	25.79%
Urban–rural income ratio	−0.093^***^	−47.56%	−0.919^*^	−469.4%	−516.9%
Personal income gap	0.010	4.870%	−0.423^***^	−216.0%	−211.2%
Total	0.288^***^	147.0%	−0.483^***^	−247.0%	−100%

* *p* < 0.05, ** *p* < 0.01, *** *p* < 0.001.

#### 5.4.1 Objective social status

Since 2010–2013, the objective social status of the youth has improved significantly. The education level, occupational status and income has increased continuously, but apart from occupational status, the positive effect of other objective social status variables on confidence in future development has significantly decreased. According to Table 4, for the structure effect of years of education, the difference value is 0.072, and the contribution rate is 36.67%. That is, 0.367 times the absolute value of the total variance in youth confidence in development (0.196). It indicates that, in the absence of an increase in average years of education, youth confidence in development would be lower than it currently is, specifically decreasing by 0.072. For the composition effect, the difference value is −0.261, and the contribution rate is −133.4%. That is, −1.334 times the absolute value of the total variance in youth confidence in development (0.196). It indicates that, if the impact of education on confidence in development has not decreased, youth confidence in development would be higher than it currently is, potentially reversing the so-called “paradox of confidence in development,” specifically increasing by 0.261. The total contribution rate of the effect is −96.68%, which means that although the increase in the average years of education has enhanced youth confidence in development, the decrease in the impact degree has significantly offset the aforementioned benefits, ultimately making the educational factor a significant constraint on the improvement of youth confidence in development. Similarly, income is also a significant factor limiting the enhancement of confidence in development, and the total contribution rate of the effect is −128.8%, mainly comes from composition effect (−129.8%). This means that if the positive effect of income on the confidence in development has not declined, youth confidence in development in 2018–2021 would become higher than that in 2010–2013. For the total effect of income, the contribution rate is −57.95%, that is, it also shows a similar impact to that of education and income.

Evidently, in the influence of objective social status on the changes in youth confidence in development, the composition effect is dominant, and the contribution rate of the total effect is also negative. This indicates that despite significant improvements in the education level, income, and occupational status of the youth, objective social status is no longer the primary factor determining youth confidence in development in the new era. The decrease in the extent of this influence is a key reason restricting the enhancement of youth confidence in development.

#### 5.4.2 Subjective social comparison

Since 2010, people’s sense of upward mobility has declined slightly but their sense of fairness has increased significantly, and the impact degree of both on the confidence in development has generally declined. According to Table 4, the reduced sense of upward mobility (structure effect) significantly reduces youth confidence in development, and the contribution rate of this part is 18.70%, while the contribution rate of its composition effect is only 1.55%. The effect of rising sense of social fairness (structural effect) is more obvious, providing a gain of 32.8%, indicating that if the sense of social fairness does not rise, then youth confidence in development in the new era will be 0.064 lower than the reality. However, due to the diminished positive effect of the sense of social fairness, the contribution rate of this composition effect is -32.03%, which offsets the gains from the structure effect, resulting in an overall contribution rate of only 0.77% for the sense of social fairness. Therefore, in terms of changes in youth confidence in development, the overall contribution provided by subjective social comparison is not high. Among these, the sense of social fairness does not exert a significant influence on the changes in youth confidence in development. The decrease in the sense of upward mobility slightly contributes to the reduction of youth confidence in development, but it is not the main cause of the “paradox of confidence in development”.

#### 5.4.3 Macro social environment

Since 2010–2013, there has been a significant improvement in regional economic development levels, and its impact on youth confidence in development has also significantly increased. So, how does this change affect the changes in youth confidence in development? According to Table 4, for the economic development level, the contribution rate of the structure effect is 98.21%, the contribution rate of the composition effect is 61.89%, and the contribution rate of the total effect is 160.11%. This shows that the enhancement of economic growth and its positive impact is a key factor in determining the changes in youth confidence in development, and has played an important role in improving it. Without the above changes, youth confidence in development in 2018–2021 would be 0.313 lower than now, that is, 1.6 times the absolute value of the existing gap would be reduced.

Since 2010–2013, although housing prices have continued to rise, the pressure from the living cost, with house-price-to-income ratio as an indicator has actually decreased slightly. According to Table 3, for the house-price-to-income ratio, the contribution rate of the structure effect is 36.08%, which indicates that the decreased house-price-to-income ratio has played a role in improving youth confidence in development. But the contribution rate of the composition effect is −10.29%, that is, the enhancement of the negative impact of the house price-to-income ratio (although it is not significant) offsets the gain brought by the decline in the house-price-to-income ratio to a certain extent. Finally, the contribution rate of the total effect from the house-price-to-income ratio is −25.79%, which is not the decisive factor leading to the changes in youth confidence in development.

In 2010–2013, the income gap between urban and rural areas was an important factor to improve youth confidence in development. However, from 2010 to 2021, the urban–rural income ratio significantly reduced, and its positive effect on youth confidence in development was also significantly reduced and no longer significant. According to Table 4, for the urban–rural income ratio, the contribution rate of its structure effect is −47.56%, the contribution rate of its composition effect is −469.4%, and the contribution rate of its total effect is −516.9%. This shows that the decrease of urban–rural income ratio and its influence degree is the key factor leading to the decrease of youth confidence in development, especially its composition effect. If the positive impact of urban–rural income ratio on confidence in development has not decreased, then youth confidence in development in 2018–2021 would be 0.919 higher than that now, almost one level higher.

Since 2010–2013, the personal income gap has widened significantly, and its impact on confidence in development has shifted from positive to negative, which has had an important impact on the changes in youth confidence in development over the past decade. For personal income gap, the contribution rate of its structure effect is 4.87%, and its impact is not significant. However, the contribution rate of its composition effect reached −216%, and the contribution rate of its total effect reached −211.2%. This shows that the impact of the personal income gap on the changes in youth confidence in development mainly comes from the composition effect. If the impact of the personal income gap has not changed from positive to negative, youth confidence in development in 2018–2021 would be 0.423 higher than that now.

In summary, how did the phenomenon of declining youth confidence in development in the new era come into being? Based on the contribution rate of each factor and the significance of its different effects, we find that the objective social status and macro social environment are the decisive factors that cause this “paradox of confidence in development.” The composition effect of the two, or the changes of the formation mechanism of confidence in development is the main mechanism for the decline of the confidence in development. Among them, the driver to enhance confidence in development mainly comes from three points: the economic development level and its positive impact, the increase of average years of education, and the decline of house-price-to-income ratio. The driver of reducing confidence in development mainly comes from three points: the positive impact of education and income on confidence in development is reduced, the urban–rural income ratio and its positive impact are reduced, and the impact of personal income gap is changed from positive to negative. In the confrontation between the two kinds of drivers, the driver to reduce the confidence in development (mainly based on the composition effect of education, income and income gap) overwhelms the driver to enhance the confidence in development (mainly based on the structure effect and composition effect of economic development level), resulting in the decline of youth confidence in development.

## 6 Conclusion

Sociology came into being under the background of rapid social change in modern times. It is one of the core theoretical propositions of sociology to explore the evolutionary process of social change and to decipher the profound influence of social change. This profound social change is not only reflected in the fields of production modes, and political systems, etc., but also in the fields of cultural concepts such as values and social attitudes. Among the studies of these cultural concepts, the study of confidence in development is a rare and future-oriented research scope with unique academic value, but existing studies still do not pay enough attention to this aspect.

In the past 3 years, the changes unseen in a century have been intertwined with the pandemic of the century, significantly increasing social uncertainties. Looking forward to the future, in the context of the still complex international situation, boosting confidence in development and enhancing psychological expectations are of great significance for promoting the healthy development of social mentality and restoring economic growth. As the hope of the country and the future of society, the mental health of young people is particularly important for assessing the social development situation and promoting economic and social development. Therefore, this paper uses the data from China General Social Survey (CGSS) from 2010 to 2021, takes youth confidence in development as the indicator of youth mental health, and analyzes the latest situation, changing trend and formation mechanism of youth confidence in development, which has important theoretical significance and practical value. It is of great importance to promote the development of relevant theories and youth mental health.

Research has found that, firstly, Chinese youth in the new era are relatively optimistic about their future development and have strong confidence in development. Specifically, from 2018 to 2021, the average confidence in development of young people was 5.892, and an upper middle level of confidence in future development was dominant. Nearly 60% of young people believed that they would surpass the social average level 10 years later.

Secondly, in terms of the changing trend, a “paradox of confidence in development” has emerged: With the continuous and stable growth of the national economy and the continuous improvement of people’s living standards, and with more positive trends in indicators such as years of education for young people, their income, occupational status, and even their sense of social fairness, the house-price-to-income ratio, and the urban–rural income gap, compared with the period from 2010 to 2013, youth confidence in future development has not increased. Instead, it has slightly declined.

Thirdly, the formation mechanism of youth confidence of in development has undergone obvious changes: The influence of objective social status on confidence in development has significantly decreased, while the influence of macro social environment on confidence in development has greatly increased. In other words, youth confidence in future development no longer depends on “who I am” (personal resource endowment), but rather more on the “macro environment” (macro social environment). Particularly, from 2010 to 2013, both the urban–rural income gap and the personal income gap had a positive impact on confidence in development. However, from 2018 to 2021, the positive impact of the urban–rural income gap was no longer significant, and the impact of the personal income gap turned negative, indicating that the long-term consequences of the “tunnel effect” have begun to emerge. The income gap that has remained at a high level for a long time is no longer a factor that encourages hard work, inspires dreams, and raises expectations, but has instead started to become a hidden danger that triggers social anxiety and personal confusion.

Fourthly, this change in the formation mechanism of confidence in development (i.e., the composition effect) is the main mechanism determining the changing trend of youth confidence in development and causing the “paradox of confidence in development.” The results of the effect decomposition show that the structure effect has a relatively weak impact on the changes of confidence in development, that is, although changes such as the improvement of education, occupational status, personal income, and the economic development level, as well as the decrease in the house-price-to-income ratio, are conducive to the enhancement of youth confidence in development, they do not play a dominant and decisive role. On the contrary, the composition effect has a greater impact on the changes of confidence in development. The positive impacts of factors such as education, income, and the sense of fairness have decreased, and the positive impact of the income gap either disappears or turns negative, offsetting the gains of confidence in development brought about by structure effects such as economic growth and income improvement, and resulting in a decrease in youth confidence in development. Among them, the composition effect of the income gap plays a decisive role.

Evidently, the “paradox of confidence in development” is the result of changes in macro social environment, and it is necessary to improve youth confidence in development by regulating social structural factors. To this end, on the one hand, it is necessary to promote the reform of housing policies, adhere to the principle of “houses for living in, not speculation,” increase the supply of social housing, moderately lower real estate loan tax rates, and curb the rapid increase in housing prices, thus reducing the financial burden on people and strengthening their ability to withstand future risks. Specifically, efforts should focus on improving a balanced housing supply system that integrates both renting and purchasing, increasing the availability of affordable rental housing and shared ownership housing, optimizing the distribution of land transfer revenues, and exploring the use of collective construction land for rental housing. At the same time, property tax reform should be promoted as a key source of local government revenue to curb real estate speculation and reduce reliance on land-based financing. On the other hand, it is necessary to further narrow the income gap, continuously improve the tax system and transfer payments, promote coordinated regional development, and strengthen the urban–rural social security system. It is essential to strengthen the role of redistribution and tertiary distribution in wealth distribution, narrow the income gap, and promote common prosperity. Specifically, the progressive tax system should be improved, the proportion of direct taxes should be increased, and the scope and share of income tax collection should be optimized to prevent double taxation and under-collection. Additionally, a well-designed transfer payment policy should be implemented to ensure that low-income groups receive adequate support while maintaining the existing income ranking, thereby maximizing the effect of reducing income gaps.

Finally, this paper still has many limitations, mainly as follows: First, there is a relatively large urban–rural gap in China, and there may also be differences in the confidence in development of urban and rural youth and its formation mechanism. Second, is the changing trend of youth confidence in development from 2010 to 2021 a long-term characteristic of China’s social development or just a segment in the non-linear changes? Due to limited space and data, this paper has not fully discussed these issues, and it is hoped that follow-up studies can supplement them.

## Data Availability

Publicly available datasets were analyzed in this study. This data can be found at: http://cgss.ruc.edu.cn/.

## Footnotes

^1^If the next lane continues to move and the lane itself has been congested, it will make people more frustrated, i.e., the long-term impact of the “tunnel effect.”

^2^Data source: National Bureau of Statistics, https://data.stats.govcn/easyquery.htm?cn=C01

^3^China has a vast territory, and the phenomenon of unbalanced regional development is quite prominent. There are significant differences among regions in terms of economic development, cost of living, income gap, etc. Compared with the overall development of the country, the perception of issues such as the employment and entrepreneurship environment, housing prices, and the gap between the rich and the poor among most ordinary people still comes from their daily life experience. The radius of daily life activities rarely crosses provincial - level administrative units. Therefore, in this paper, the measurement of the macro environment is based on the province (autonomous region, municipality) where the observation is located.

^4^ https://data.stats.gov.cn

## References

[ref1] Alves W. M. Rossi P. H. (1978). Who should get what? Fairness judgments of the distribution of earnings. Am. J. Sociol. 84, 541–564. doi: 10.1086/226826

[ref2] Blinder A. S. (1973). Wage discrimination: reduced form and structural estimates. J. Hum. Resour. 8, 436–455. doi: 10.2307/144855

[ref3] Cai H. Lu Y. Zhang Y. J. (2020). House price, house property, and urban residents’ subjective socio-economic status: an empirical study based on China’s labor dynamic survey. J. Sun Yat-sen Univ. 60, 144–156. doi: 10.13471/j.cnki.jsysusse.2020.02.015

[ref4] Chen Y. S. Fan X. G. (2016). Subjective social status, income inequality and subjective perceptions of mobility (2003-2013). Soc. Sci. China 12, 109–207.

[ref5] Chen W. M. Li X. Q. (2021). The impact of class identity and social mobility expectations on fertility intentions: with a discussion on the formation mechanism of the low fertility trap. J. Nankai Univ. 2, 18–30.

[ref6] Cong Y. F. (2013). Analysis on social confidence and influencing factors of new white-collar migrants: based on the empirical investigation in Shanghai. Youth Stud. 6, 48–93.

[ref7] Curtis J. (2016). Social mobility and class identity: the role of economic conditions in 33 societies, 1999–2009. Eur. Sociol. Rev. 32, 108–121. doi: 10.1093/esr/jcv077

[ref8] Easterlin R. A. Morgan R. Switek M. Wang F. (2012). China’s life satisfaction, 1990–2010. Proc. Natl. Acad. Sci. USA 109, 9775–9780. doi: 10.1073/pnas.1205672109, PMID: 22586096 PMC3382525

[ref9] Erikson R. Goldthorpe J. H. (1992). The CASMIN project and the American dream. Eur. Sociol. Rev. 8, 283–305. doi: 10.1093/oxfordjournals.esr.a036642

[ref10] Hirschman A. O. Rothschild M. (1973). The changing tolerance for income inequality in the course of economic development: with a mathematical appendix. Q. J. Econ. 87, 544–566. doi: 10.2307/1882024, PMID: 39964225

[ref11] Lei K. C. (2015). Young people’s confidence in class status and its influencing factors. Youth Stud. 4, 9–94.

[ref12] Li W. (2019). The sense of social fairness: structure and trend—an analysis of the trend of public social fairness between 2006 and 2017. J. Huazhong Univ. Sci. Technol. 33, 110–121. doi: 10.19648/j.cnki.jhustss1980.2019.06.14

[ref13] Li J. (2021). From income to wealth: class identification and its change in urban China—a temporal trend analysis on Shanghai from 1991 to 2013. Sociol. Res. 36, 114–228.

[ref14] Li L. Liu B. (2012). The impact of anticipation on happiness of urban residents in China. Nankai Econ. Stud. 4, 53–67. doi: 10.14116/j.nkes.2012.04.002

[ref15] Li L. L. Shi L. Zhu B. (2018). Solid or fluid? Social class structure transition trends in contemporary China during the past 40 years. Sociol. Res. 33, 1–34. doi: 10.19934/j.cnki.shxyj.2018.06.001

[ref16] Li L. L. Wang Y. C. (2020). Social attitude change in China: general tendency and the influence mechanism (2005-2015). Open Times 6, 129–145.

[ref17] Li H. L. Wei Q. G. (2013). Research on social prosperity and social confidence. Beijing: China Social Science Press.

[ref18] Li L. L. Zhu B. (2015). Changes in the pattern of intergenerational mobility in contemporary China. Soc. Sci. China 5, 40–204.

[ref19] Lindemann K. Saar E. (2014). Contextual effects on subjective social position: evidence from European countries. Int. J. Comp. Sociol. 55, 3–23. doi: 10.1177/0020715214527101

[ref20] Liu J. M. (2006). Expansion of higher education in China and inequality in entrance opportunities: 1978-2003. Chin. J. Sociol. 3, 158–209. doi: 10.15992/j.cnki.31-1123/c.2006.03.009

[ref21] Liu C. (2016). Urban youth’s social confidence and its influencing factors. Youth Stud. 2, 11–94.

[ref22] Oaxaca R. (1973). Male-female wage differentials in urban labor markets. Int. Econ. Rev. 14, 693–709. doi: 10.2307/2525981

[ref23] Parkin F. (1974). “Strategies of social closure in class formation” in The social analysis of class structure. ed. Parkin F. (London: Routledge), 1–18.

[ref24] Piketty T. (2014). Capital in the Twenty-First Century. Beijing: CITIC Press Group.

[ref25] Su Y. H. (2019). The temporal dimension of contemporary Chinese youth’s career development expectation. China Youth Stud. 10, 12–18. doi: 10.19633/j.cnki.11-2579/d.20190924.005

[ref26] Veblen T. B. (2017). The theory of the leisure class: An economic study of institutions. Beijing: China Renmin University Press.

[ref27] Xu Q. He G. Y. Hu J. (2020). Marketization and change of perceptions about distributive justice in China: 2005-2015. Chin. J. Sociol. 3, 88–116. doi: 10.15992/j.cnki.31-1123/c.2020.03.004

[ref28] Zhang Y. Wei Q. G. Li H. L. (2015). Social prosperity and social confidence in the developmental process—constructing concepts, scales and indices. Soc. Sci. China 4, 64–206.

[ref29] Zhao D. L. Yuan Y. (2019). Class differences in people’s future life expectations in the new era and the mediating effect of the sense of social justice. J. Harbin Inst. Technol. 21, 54–62. doi: 10.16822/j.cnki.hitskb.2019.03.029

[ref30] Zhou J. Yu B. (2015). Future expectation and household asset allocation affected by social insurance—empirical study based on the survey Shanghai residents. Collect. Essay. Fin. Econ. 9, 27–33. doi: 10.13762/j.cnki.cjlc.2015.09.004

[ref31] Zhu L. (2013). Social psychological interpretation of public confidence dispersion. Beijing: Tongfang Knowledge Network Technology Co., Ltd., 10–12.

